# Examining the Prospective Bidirectional Associations between Subjective and Objective Attractiveness and Adolescent Internalizing Symptoms and Life Satisfaction

**DOI:** 10.1007/s10964-022-01700-7

**Published:** 2022-11-12

**Authors:** Natasha R. Magson, Ella L. Oar, Jasmine Fardouly, Ronald M. Rapee, Justin Y. A. Freeman, Cele E. Richardson, Carly J. Johnco

**Affiliations:** 1 Centre for Emotional Health, Macquarie University, Sydney, Australia; 2 School of Psychology, UNSW Sydney, Sydney, Australia; 3 School of Psychological Science, University of Western Australia, Perth, Australia

## Abstract

Research has consistently shown that more physically attractive individuals are perceived by others to be happier and better psychologically adjusted than those perceived as less attractive. However, due to the lack of longitudinal research in adolescents, it is still unclear whether poor mental health predicts or is predicted by either objective or subjective attractiveness during this critical developmental period. The purpose of the current study was to examine prospective bidirectional associations between both subjective and objective ratings of attractiveness, life satisfaction and symptoms of social anxiety, depression and eating disorders (i.e., internalizing symptoms) from early to mid-adolescence. Participants (T1: *N* = 528, 49.9% girls; *M*_*age*_ = 11.19; *SD* = 0.55) were followed annually over four time points. The cross-lagged panel model results revealed evidence of prospective associations between both forms of attractiveness and life satisfaction and internalizing symptoms, which were driven more by changes in the mental health outcomes than by changes in the subjective and objective attractiveness ratings. The results also indicated that the pattern, strength, and direction of the associations tested were robust across boys and girls, and white and non-white ethnic groups. Overall, the findings suggest that it is important to find effective ways of educating adolescents who are unhappy with their appearance that making changes to improve their mental health, rather than focusing on their physical appearance, will have benefits not only for how they perceive themselves but also for how they are perceived by others.

## Introduction

Research has consistently shown that more physically attractive individuals are perceived by others to be better psychologically adjusted, happier, more intelligent, and more socially competent than those perceived as less attractive (Rennels, 2012). This has been termed the “attractiveness halo effect” (Dion et al., 1972) whereby people attribute other socially desirable traits and qualities (i.e., success, intelligence, happiness) to those they perceive to be physically attractive. Although there is an abundance of evidence supporting the halo effect, research has inconsistently examined whether physical attractiveness is associated with actual mental health and wellbeing outcomes, and importantly, whether self-perceptions of attractiveness (subjective) are more important for enhancing these outcomes than ratings of attractiveness by others (objective). Of the research that has investigated the link between objective and subjective attractiveness and psychological outcomes, much of it is outdated, cross-sectional, unidirectional, and has been conducted in adult samples. As the internalization of socially sanctioned beauty ideals occurs during early adolescence (Lawler & Nixon, 2011), and adolescence is a particularly vulnerable period for the onset of internalizing disorders (Rapee et al., 2019), more research investigating the links between objective and subjective attractiveness and internalizing symptoms in youth is imperative to inform prevention and early intervention.

The current study sought to address these research gaps by investigating whether objective and/or subjective physical attractiveness were prospectively associated with life satisfaction and internalizing symptoms from early to mid-adolescence. Although studies investigating internalizing symptoms typically focus on depression and anxiety, the development of eating disorders shows very strong similarities to the development of anxiety and depression, as they share common risk and maintenance factors (Sander et al., 2021), commonly co-occur (Blinder et al., 2006), and are most likely to first emerge during the adolescent period (Rapee et al., 2019). The three disorders have also been grouped together in theoretical models of social-emotional distress (Rapee et al., 2019) and empirically derived classification systems based on interrelated symptom manifestations of internalizing psychopathology (Kotov et al., 2017). Therefore, the current study measured all three forms of internalizing distress via a latent factor that modelled common variance indicated by symptoms of depression, social anxiety, and eating pathology.

### Attractiveness and Links to Mental Health

The direction of the association between subjective and objective attractiveness and mental health outcomes has not yet been systematically researched, despite being predicted by a range of theories. For example, socialisation and social expectancy theories such as Status Characteristics Theory (SCT; Berger et al., 1977) assert that physical attractiveness is an observable status characteristic, much like age, sex, and ethnicity, that people use to form expectations about the competence of others. SCT maintains that those with high status characteristics (e.g., male, attractive) are perceived by others to have more influence and be more competent than those with low status characteristics (e.g., female, unattractive), particularly in social situations (Rennels, 2012). According to SCT, and social expectation theories generally, physically attractive individuals should experience better mental health because over time they internalize the positive judgements and preferential treatment of others, positively influencing attractive individuals’ self-perceptions, thoughts, and behaviors (Langlois et al., 2000). Hence greater attractiveness should predict better mental health and life satisfaction in the future.

Theory also predicts a link between self-perceptions of one’s own attractiveness and mental health. For example, the socio-cultural tripartite theoretical model (e.g., Thompson et al., 1999) suggests that our appearance-focused culture has resulted in the internalization of unrealistic and unattainable societal beauty ideals. The internalization of these ideals can create a discrepancy between one’s actual and ideal appearance, which depending on the degree of internalization, can result in body dissatisfaction, low self-esteem, and consequent increases in internalizing symptoms (Lawler & Nixon, 2011). Hence, one’s self-perceived attractiveness can be influenced by factors quite separate from their actual physical attributes, often resulting in inconsistencies between subjective and objective physical attractiveness ratings and subsequently leading to greater emotional distress.

While the above theories suggest a unidirectional association from subjective and objective attractiveness to mental health, a range of clinical theories of internalizing disorders suggest that the relationship may also operate in the opposite direction. Theories of depression point to the importance of positive reinforcement and social interactions in forming self-perceptions (Lewinsohn et al., 1980) and negative cognitive biases (Orchard and Reynolds, 2018). These cognitive biases can negatively affect perceptions of the self, including self-perceptions of attractiveness (Van de Vliet et al., 2002) which may serve to undermine any protective effect of others preferential treatment of attractive individuals. Further, social anxiety is primarily characterized by concerns about public scrutiny and negative evaluation in social situations (Spence & Rapee, 2016) and evidence shows that socially anxious individuals often perceive themselves to be less attractive when compared to ratings from others (Rapee & Abbott, 2006).

While much of the theory in this area has focused on internalizing symptoms in terms of anxiety and depression, lower perceptions of attractiveness may also lead to a range of dieting and related efforts to change appearance, in turn increasing risk for eating disorders (Reynolds & Meltzer, 2017). In addition, in those with eating pathologies, self-evaluations of attractiveness tend to be overly influenced by body shape and weight concerns and sufferers often judge themselves as less unattractive than objective observers (Cash & Deagle, 1997), again suggesting a possible bidirectional relationship. Therefore, the repeated positive social reinforcement and lack of negative evaluation provided to those who are more attractive may protect against internalizing symptoms (Feingold, 1992). Conversely, internalizing disorders may have characteristics and symptoms that in turn make a person appear less attractive to others. For example, depression is often associated with poor self-care (DiMatteo et al., 2000) and a lack of positive social skills (Verboom et al., 2014), eating pathology is characterised by significant weight loss or gain (Micali et al., 2014), and social anxiety can be associated with self-handicapping behaviors such as poor eye contact, quiet voice, and closed posture (Howell et al., 2016).

Finally, the link between attractiveness and internalizing symptoms may also differ as a function of sex, as it is well established that there are marked sex differences in the prevalence of internalizing disorders (Altemus et al., 2014) and socially sanctioned beauty ideals (e.g., thinness for girls versus muscularity for boys; Lawler & Nixon, 2011) that typically emerge during adolescence. Girls have been found to internalize socially prescribed body ideals more than boys, and feel a greater pressure to adhere to them (Knauss et al., 2008). Objectification theory (Fredrickson and Roberts, 1997) has also been used to describe how women’s early socialization and sexual objectification experiences translate into mental health difficulties, particularly internalizing disorders such as depression and eating disorders (Moradi & Huang, 2008). Further, girls are also more likely to engage in appearance-focused conversations with friends and be the target of appearance-based victimisation (Lawler & Nixon, 2011). Therefore, it is possible that the links between both subjective and objective attractiveness and mental health outcomes are stronger in girls than boys. In sum, theoretical predictions suggest that either, or both, subjective and objective attractiveness may be a result of, or lead to low life satisfaction and internalizing disorders, and that these associations may be stronger in girls.

### Attractiveness and Mental Health in Adolescence

Positive stereotypes about attractive individuals may particularly resonate with adolescents, increasing their risk for internalizing disorders. For example, research shows that appearance concerns increase markedly during early adolescence, which coincides with the cognitive, social and physical changes associated with the onset of puberty (Stice, 2003). Further, appearance-focused conversations with peers and appearance-based peer victimization are common in adolescence, and can consolidate unattainable societal beauty norms and ideals (Lawler & Nixon, 2011).

Early to mid-adolescence also marks a period of vulnerability for the onset of a particular subset of internalizing disorders such as social anxiety, eating pathology, and depression, which have been collectively referred to as “social-emotional disorders” (Rapee et al., 2019). Finally, the documented decreases in life satisfaction and self-esteem from early to mid-adolescence (Goldbeck et al., 2007), may also make adolescents particularly vulnerable to stereotypes linking attractiveness and happiness. These characteristics unique to adolescence suggest that early to mid-adolescence (pre to post puberty) may be an important developmental period in which to examine the prospective bidirectional associations between physical attractiveness, internalizing symptoms, and life satisfaction. Despite this, there has been surprisingly little research, especially longitudinally, that assesses life satisfaction and internalizing symptoms in relation to both objective and subjective aspects of attractiveness, and none that measured all of these factors bidirectionally in an adolescent sample.

### Empirical Research Investigating Objective Attractiveness and Mental Health

Most of the research examining the links between objective attractiveness and psychological outcomes in adolescents has been correlational, and results have been relatively inconsistent. Further, much of this research was carried out more than two decades ago, and with few exceptions, has not been revisited. This is surprising considering the rise of social media and adolescents’ increased exposure to not only attractive celebrities but also the abundance of attractive everyday people flooding their news feeds as popular and financially successful “influencers” (Khamis et al., 2016). This increased exposure to ordinary people reaching “celebrity” status based on their appearance may perpetuate existing stereotypes surrounding the social benefits of attractiveness to which adolescents may be particularly vulnerable due to their developing self-concept and heightened social sensitivity (Somerville, 2013). Despite this, the association between attractiveness and adolescent mental health has received very little attention in the current digital appearance-focused climate.

Some early research found that objectively-rated facial attractiveness was associated with better overall psychosocial functioning in pre-adolescents, although specific associations with depression and anxiety were not statistically significant (Perkins & Lerner, 1995). However, other research has found no significant associations between objective attractiveness and self-worth/esteem, anxiety, or depression in early adolescence (Lerner et al., 1991). Similar inconsistency can be found in research examining social anxiety and objective attractiveness, with one early meta-analysis reporting non-significant concurrent associations (e.g., Feingold, 1992), whereas a later meta-analysis including both correlational and longitudinal evidence found significant positive associations (Langlois et al., 2000). However, both meta-analyses included mostly adult samples and when younger samples were included, adolescent subgroup results were not reported making it difficult to determine whether these results were replicated in adolescents. Finally, the associations between objective attractiveness and eating disorders have been largely overlooked. This is surprising considering the large literature linking body dissatisfaction and the risk of eating disorders (e.g., Stice, 2002). Although not examining eating disorders specifically, one study found that objectively-rated facial attractiveness was associated with greater weight preoccupation in young women (Davis et al., 2000), with the authors concluding that this was due to physically attractive individuals feeling greater pressure to maintain their attractiveness. Although this study was also correlational and has not been investigated among adolescents.

The existing research reviewed above provides several hints that objectively-rated attractiveness may have associations with life satisfaction and internalizing symptoms in adolescents, but the evidence is far from consistent. Further, the rarity of longitudinal evidence means that the direction of the associations cannot be determined. Of the few longitudinal studies conducted, one found that objectively rated attractiveness based on high school yearbook photographs significantly predicted greater well-being and lower depression in late adulthood (Gupta et al., 2016). Further, a two-wave prospective study found that higher teacher, parent, and peer rated attractiveness in Grade 3 significantly predicted lower levels of depression in girls in Grade 6 (Cole et al., 1997). Although providing some evidence for a prospective association between objective attractiveness and mental health, the longitudinal evidence above is limited by only examining the unidirectional association from objective attractiveness to mental health, and by utilizing only two waves of data. Thus, to date, there has been no longitudinal research specifically examining these bidirectional changes across the critical period from early to middle adolescence.

### Empirical Research Investigating Subjective Attractiveness and Mental Health

The empirical evidence relating to the associations between subjective attractiveness and mental health is much more consistent and well supported by the larger literature on body image and mental health (e.g., Walker et al., 2018). Correlational research specific to physical attractiveness and mental health in adolescence has consistently found that high subjective attractiveness is associated with less generalized anxiety and greater self-worth (Jovanovic et al., 1989), lower social anxiety (Neto, 1993) and depression (Raible-Destan et al., 2021), less loneliness (Moore and Schultz, 1983), higher life satisfaction (Neto, 1993), and fewer eating disorder symptoms (Smink et al., 2018). What remains unclear is the direction of the association as most of the research in this area has been cross-sectional and/or has investigated subjective attractiveness as a predictor of adolescent mental health outcomes (e.g., Ehlinger & Blashill, 2016).

However, it is equally plausible that the negative cognitive biases associated with internalizing difficulties (Orchard & Reynolds, 2018) alter adolescents’ perceptions of their appearance. While there is some correlational evidence that people with internalizing problems rate themselves as less attractive than their counterparts (Rapee and Abbott, 2006), there is very little longitudinal research assessing the direction of this association. One of the few studies examining these associations bidirectionally, found that depressive symptoms predicted lower subjective attractiveness 6-months later over three successive years spanning pre to early adolescence, however, there was little support for the opposite direction of association (Cole et al., 1998). In contrast, although not assessing subjective attractiveness specifically, a longitudinal study with adolescents at ages 13, 15, 18 found that poor body image served as an antecedent to depressive symptoms, but found no evidence of a prospective association from depression to body image (Holsen et al., 2001). Thus, it remains unclear whether subjective attractiveness is predictive or reflective of mental health difficulties and to date, this question has only been addressed by a very small number of relatively old studies.

### Potential Group Differences

#### Sex

The inconsistent findings across studies and the lack of bidirectional longitudinal research is further complicated when examining sex differences. Research examining sex differences in the associations between attractiveness and internalizing symptoms in adolescence is scarce and the results mixed. Although yet to be examined in relation to adolescent objective attractiveness, research examining sex differences in the associations between subjective attractiveness and mental health in adolescents has shown that the association is either similar for both sexes (Raible-Destan et al., 2021), evident only in boys (Cole et al., 1997) or significantly stronger in girls (Cole et al., 1998). Although theory would predict that the associations between attractiveness and mental health should be stronger in adolescent girls than boys, the empirical research suggests these associations are far more complex and require further investigation.

#### Ethnicity

It is widely believed that perceptions of beauty are culturally defined rather than there being a universal standard of beauty (Yan & Bissell, 2014). However, this belief is generally not supported by the literature, which shows that beauty ideals are more similar than different across cultures and ethnic groups (Langlois et al., 2000; Yan & Bissell, 2014). It is argued that the unique and varied attractiveness standards previously found in non-Western countries, have become blurred and unified over time through the mass globalization and promotion of Western beauty ideals (Isa & Kramer, 2003). Supporting cultural assimilation to a universal attractiveness standard, numerous studies have reported high levels agreement among raters both within and between ethnic groups when evaluating the attractiveness of others (e.g., Kočnar et al., 2019), although it is important to note that others have argued that some small regional nuances remain (Marcinkowska et al., 2019). Further, the attractiveness halo effect has been documented in both Western and non-Western countries with one study demonstrating that in 45 countries across all 11 regions of the world, male and female attractive faces were perceived by raters as more emotionally stable than those judged as less attractive (Batres & Shiramizu, 2022). However, there does not appear to be any research examining cross-cultural differences in the associations between attractiveness and *actual* mental health and well-being outcomes. Hence, this will be examined in the current research.

## The Current Study

Most of the research examining the associations between attractiveness and psychological outcomes is outdated and has been limited to observer perceptions of mental health, cross-sectional designs, unidirectional or correlational analyses, and adult samples. To address these gaps, the aim of the present longitudinal study was to determine whether more attractive adolescents, as rated by the self and others, experienced greater life satisfaction and lower internalizing symptoms than those rated as less attractive. As prior research also shows that individuals experiencing elevated internalizing symptoms and lower life satisfaction perceive themselves as less attractive, and are rated as less attractive by others, the opposite associations were also tested, that is, whether changes in life satisfaction and internalizing symptoms were associated with prospective changes in subjective and objective attractiveness. Based on previous findings, it was predicted that subjective attractiveness would have more robust and reliable prospective associations with internalizing and life satisfaction outcomes than objective attractiveness, however, the direction of these associations was not predicted a priori due to the lack of previous research examining these longitudinally and bidirectionally. The second aim of the study was to determine whether the associations differed by sex and ethnicity, however, as previous research is relatively inconsistent in terms of sex effects and completely lacking in relation to ethnic differences, this aspect of the study was exploratory, and no specific hypotheses were formulated.

## Method

### Participants and Procedure

Participants were part of the Risks to Adolescent Wellbeing (RAW) Project. After receiving the necessary ethics approval, a convenience sample was recruited through social media advertisements, school newsletters, and flyers distributed throughout the community. Both the adolescent and the primary caregiver provided written consent each year. As part of the larger study, adolescents and their primary caregiver completed an online questionnaire and a diagnostic phone interview. Adolescents also completed a two-hour laboratory session. The full list of annual measures (along with the de-identified data and code used in the analyses) can be found in the OSF repository here: https://osf.io/drsym/?view_only=bc0fa561ec4d4c4d9fa29f2f311ff8b9, and those pertaining to the current study are presented in the measures section below. Sample size was ultimately determined by the number of participants that could be recruited over the 12-month period from August 2016 to August 2017, although a power analysis calculation (Soper, 2022) indicated that a minimum sample of 100 participants was required to sufficiently test the proposed internalizing CFA measurement model and a minimum of 342 participants were required to detect a small to medium effect (0.2 - 0.5) in the cross-lagged panel models estimated. In aiming to increase power, only well-validated measures of the mental health outcomes were used, and the interrater reliability of objective ratings of attractiveness were evaluated prior to analyses.

At Time 1 (T1), the final sample included 528 Grade 6 students and their primary caregiver (96% mothers) residing in Australia. The participants were predominately born in Australia (90.6%), spoke English as a first language (96.4%), and reported a middle to high socioeconomic status (80%). The majority of the sample identified as white (81.9%), the remainder identified as Asian (6.4%), Middle Eastern (1.5%), or other (10.2%; Eurasian 4.4%, European 2.1%, The Americas 1.3%, Indian 1.1%, Maori/Islander 0.9%, unknown 0.4%). The current study comprises data from participants who had responded to the variables of interest on at least one occasion with an overall dropout rate of 17.8% from T1 to T4. The sample included 528 participants at T1 (49.9% girls; *M*_*age*_ = 11.19; *SD* = 0.55), 495 at T2 (48.4% girls; *M*_*age*_ = 12.67; *SD* = 0.56), 476 at T3 (48.1% girls; *M*_*age*_ = 13.70; *SD* = 0.55), and 434 at T4 (48.5% girls; *M*_*age*_ = 14.80; *SD* = 0.53). Two of the participants at T3 and T4 indicated that their gender was neither male nor female. For participation in the research, families were remunerated with an AUD$100 gift voucher each year, and adolescent participants also received a small gift bag valued at approximately $20 to $30.

### Measures

#### Demographics

Youth completed a demographic questionnaire which included: biological sex (male/female), gender (male, female, other: please specify, or prefer not to answer), main language spoken at home, country of birth, and ethnicity (White, Indigenous, Middle Eastern, Asian or other: please specify). The primary caregiver also provided annual household income.

#### Subjective Attractiveness

Adolescents were asked to “rate how attractive (good looking) you think each of the following parts of your appearance are: your face, your hair, your body, and your overall appearance” on a Likert scale from 1 (*very unattractive*) to 5 (*very attractive*). Responses across the four items were averaged to create an overall subjective attractiveness score ranging from 1 to 5. The reliability of the measure was acceptable across the four time points (T1 α = 0.87, T2 α = 0.87, T3 α = 0.86, T4 α = 0.84).

#### Objective Attractiveness

At each time point, 10 independent raters, who were volunteer psychology undergraduate or honours students, scored the attractiveness of the adolescents based on two full body color photographs taken by the experimenter during the in-person laboratory sessions at each time wave. One photograph was of adolescents in a neutral pose (i.e., passport style facial expression, arms by their sides, and feet together) and one photograph was of them in an animated pose of their choosing (i.e., participants were instructed to pose as they would for a social media post). As with the subjective measure above, the raters were asked to score the attractiveness of participant’s face, hair, body, and overall appearance on a scale from 1 (*very unattractive*) to 5 (*very attractive*). Mean scores were calculated for each photograph by averaging scores across the four items. The attractiveness ratings of the neutral and animated photographs were found to be highly correlated (T1 = 0.90, T2 = 0.91, T3 = 0.93, and T4 = 0.90) so scores were averaged across the two photographs to create a single objective attractiveness mean score at each measurement occasion. Based on the values recommended Koo and Li (2016), at each time point the consistency of ratings between raters was excellent (ICC_*2,10*_ = 0.93, 0.90, 0.92, and 0.91, T1 to T4, respectively) and the level of absolute agreement across all raters was good to excellent (ICC_*2,10*_ = 0.91, 0.88, 0.88, and 0.90, T1 to T4, respectively). Raters varied across years, however, reflecting the broader psychology student demographic, most were female (95%), aged between 20–25 years (90%), and born in Australia (90%). The raters self-identified their ethnicity as white (61.1%), Indian (16.7%), Asian (11.1%), and African (11.1%).

#### Internalizing Symptoms

Internalizing symptoms were measured using a latent factor indicated by three observed variables comprising symptoms of depression, social anxiety, and eating pathology. Depressive symptoms over the past two weeks were measured using the 13-item Short Mood and Feelings Questionnaire-Child (SMFQ; Angold et al., 1995). Participants indicated how true each statement (e.g., “I felt miserable or unhappy”) was for them on a 3-point scale (0 = *not true*, 1 = *sometimes true*, 2 = *always true*) and scores were summed across all items to create a total score ranging from 0 to 26. The SMFQ is not a diagnostic tool, although the original authors recommend using a cut-off score of 8 to indicate elevated symptoms of depression. The number of participants meeting this criterion in the current study were: 105 (19.9%) at T1, 76 (14.4%) at T2, 90 (17%) at T3, and 129 (24.4%) at T4. The measure demonstrated good reliability across all time points in the current study (T1 α = 0.88, T2 α = 0.89, T3 α = 0.92, and T4 α = 0.94).

Social anxiety symptoms were measured with the 6-item social anxiety subscale of the Spence Children’s Anxiety Scale (SCAS-C; Spence et al., 2003). Participants rated how often each of the items listed (i.e., “I worry what other people think of me”) happened to them on a scale of 0 (*never true*) to 3 (*always true*). Scores were summed to create a total score, with possible scores ranging from 0 to 18. Although not a diagnostic instrument, it is recommended that a cut off score of 8 for girls and 10 for boys be used to indicate elevated symptom levels of social anxiety requiring further review. In the present sample, 79 (15%) met that criterion at T1, 70 (13.3%) at T2, 73 (13.8%) at T3, and 100 (18.9%) at T4. In the current study, the Cronbach alpha values at each time point were acceptable (T1 α = 0.76, T2 α = 0.78, T3 α = 0.78, T4 α = 0.82).

Finally, eating pathology symptoms were measured using the 26-item Children’s Eating Attitude Test (chEAT; Maloney et al., 1988). Participants responded to each statement (e.g., “I feel very guilty after eating”) on a 6-point scale (0 = *never*, 0 = *rarely*, 0 = *sometimes*, 1 = *often*, 2 = *very often*, 3 = *always*), and after reverse coding the relevant items, all responses were then summed to form a total score ranging from 0 to 78. For screening purposes only, the authors of the chEAT recommend using a cut-off score of 20 to indicate the presence of disturbed eating patterns that should be assessed further with formal diagnostic tools. In the current study, this criterion was met by 11 participants (1.9%) at T1, 11 (2.1%) at T2, 10 (1.9%) at T3, and 20 (3.8%) at T4. The Cronbach alpha values indicated that the internal consistency of the measure was good (T1 *α* = 0.83, T2 *α* = 0.85, T3 *α* = 0.88, T4 *α* = 0.91).

#### Life Satisfaction

The 9-item Student Life Satisfaction Scale (SLSS; Huebner, 1994) measures global life satisfaction in school-aged children. Participants indicated their level of agreement with each statement (e.g., “I have a good life”) on a 7-point scale ranging from 1 (*strongly disagree*) to 7 (*strongly agree*). After reverse coding the two negatively worded items, item scores were averaged to provide an overall mean score (range 1–7) with higher scores indicating greater life satisfaction. The Cronbach alpha values for the measure in the current study were good at all four time points (T1 α = 0.90, T2 α = 0.92, T3 α = 0.93, and T4 α = 0.92).

### Statistical Analysis

SPSS Statistics version 27 (IBM Corp. (2021)) was used for data screening, assumption testing, the creation of subscales, and the calculation of all descriptive statistics. The main analyses were carried out in Mplus version 8.0 (Muthén & Muthén, 2017) using the maximum likelihood estimator (MLR) to account for any non-normality of the data, and full information maximum likelihood (FIML) estimation to handle missing data. A series of four-wave cross-lagged panel models (CPLMs) were used to evaluate the reciprocal relations between objective and subjective attractiveness, internalizing symptoms, and life satisfaction. Prior to running the full cross-lagged panel models, a confirmatory factor analysis (CFA) in Mplus was carried out to confirm the relations between the internalizing symptoms latent construct and its corresponding observed indicators comprising symptoms of depression, social anxiety, and eating pathology. There were an insufficient number of participants in each of the ethnic categories to justify making comparisons, therefore for analytic purposes, all participants identifying as other than white were collapsed into a single “non-White” group and were compared to the “White” group. Further, to ensure that the latent internalizing factor was equivalent for boys and girls, and the White and non-White groups, sequential multi-group confirmatory factor analysis was also conducted in Mplus testing the equivalence of the latent factor across sex and ethnicity at three levels of invariance. Following the guidelines of Geisinger and McCormick (2013), the model was first estimated simultaneously for both groups (i.e., boys and girls; White and non-White) allowing all parameters to vary (configural invariance). Next equality constraints were placed on the factor loadings (metric invariance), and then the item intercepts (scalar invariance) to justify making mean comparisons across sex and ethnicity. As recommended by Cheung and Rensvold (2002) a measure was considered invariant when the difference in CFI values (△CFI) between the increasingly restricted models did not exceed 0.01.

Next, CLPM models were run separately for both subjective and objective attractiveness and each mental health outcome (i.e., internalizing, life satisfaction; totalling 4 models), with all analyses controlling for sex and using a significance value of 0.05. Following the recommendations of Orth et al. (2021), all autoregressive and cross-lagged paths were constrained to be equal between measurement occasions, which did not significantly worsen model fit according to the Cheung and Rensvold (2002) criteria noted above. In addition, the residuals of each variable were correlated within time points in all models. Model fit for all estimated models (i.e. CFA, multi-group, and CLPMs) were evaluated using the comparative fit index (CFI) and root mean square error of approximation (RMSEA) with CFI values greater than 0.90 and 0.95, and RMSEA values below 0.080 and 0.050, indicative of acceptable and good model fit respectively (Hooper et al., 2008). Lastly, to test for moderation by sex and ethnicity, interaction terms were added to the models. These were created using the Mplus “define” command for observed variables (e.g., objective attractiveness x ethnicity) and the XWITH command for creating the interaction terms for sex and ethnicity and the internalizing latent factor.

## Results

### Preliminary Results

The attractiveness, life satisfaction and social anxiety variables conformed to normality assumptions whereas depression and eating pathology were positively skewed. With the exception of objective attractiveness (1.5% missing due to participants not attending the on campus lab session) there was no missing data at T1. Due to participant dropout in subsequent waves, there was between 5.9% and 6.3% entirely missing at T2 (0.04% missing due to skipped items), 9.5% to 9.8% entirely missing at T3 (0.03% missing due to skipped items), and 17.2% to 17.8% entirely missing at T4 (0.06% missing due to skipped items). Little’s MCAR test (Little, 1988) was not significant (*x*^2^ = 402.79, df = 394, *p* = 0.369) thus data was assumed to be missing at random. The Mplus coverage covariance matrix indicated that the available data across the four waves ranged from 82% to 94%.

Descriptive statistics and *t*-test results are displayed in Table 1. There were no significant sex differences in subjective or objective attractiveness ratings, however girls scored significantly higher than boys on all measures of internalizing symptoms, and boys scored higher than girls on life satisfaction at T3 and T4. There were no mean differences between White and non-White ethnic groups in subjective or objective attractiveness ratings, social anxiety, or eating pathology. The non-White group reported significantly more depression at T3 and lower life satisfaction at T2, T3, and T4. As shown in Table 2, the correlations between objective and subjective attractiveness within (*r* = 0.14 - 0.24) and across (*r* = 0.09 - 0.29) waves were small. In contrast, the correlations for objective attractiveness across waves were moderate to high (*r* = 0.55 - 0.72), whereas correlations for subjective attractiveness across waves were small to moderate (*r* = 0.32 - 0.59). Subjective and objective attractiveness showed negative correlations with each type of internalizing symptom, and positive correlations with life satisfaction. Point-biserial correlations showed that sex was significantly correlated with objective attractiveness ratings (*r’s* = 0.14 - 0.22) and social anxiety (*r’s* = 0.14 - 0.36) at each time point, and with depression, life satisfaction, and eating pathology at T3 and T4 (*r’s* = −0.23 - 0.36). Ethnicity was not associated with either form of attractiveness, and only weakly associated with depression at T3 (*r* = −0.12) and life satisfaction at T2, T3, and T4 (*r’s* = 0.09 - 0.14). The correlations for boys and girls, and white and non-white ethnic groups are presented separately in the Appendix.

**Table 1:** Observed variable means, standard deviations, range of scores, and independent t-tests of mean group differences

	Total Sample			Girls		Boys		T-test	White		Non-White		*T*-test
Variable	*N*	*M (SD)*	Range	*N*	*M (SD)*	*N*	*M (SD)*	*t-value*	*N*	*M (SD)*	*N*	*M (SD)*	*t-value*
T1 SubAtt	528	3.61 (0.80)	1–5	258	3.67 (0.85)	270	3.55 (0.76)	−1.84	432	3.61 (0.81)	96	3.62 (0.76)	−0.09
T2 SubAtt	495	3.59 (0.08)	1–5	238	3.65 (0.86)	257	3.53 (0.72)	−1.83	408	3.59 (0.80)	87	3.58 (0.74)	0.06
T3 SubAtt	476	3.49 (0.77)	1–5	229	3.52 (0.81)	247	3.46 (0.74)	−1.63	392	3.52 (0.77)	84	3.36 (0.77)	1.77
T4 SubAtt	434	3.42 (0.80)	1–5	210	3.35 (0.84)	224	3.49 (0.75)	−1.62	354	3.44 (0.79)	80	3.33 (0.83)	1.16
T1 ObAtt	520	2.95 (0.38)	1.75–4.04	255	3.03 (0.43)	265	2.87 (0.32)	−0.87	427	2.94 (0.38)	93	2.97 (0.42)	−0.65
T2 ObAtt	494	3.05 (0.34)	1.98–4.03	236	3.10 (0.33)	258	3.00 (0.34)	−0.86	407	3.05 (0.33)	87	3.05 (0.38)	−0.05
T3 ObAtt	476	2.98 (0.36)	1.85–4.10	226	3.04 (0.39)	250	2.94 (0.32)	1.90	391	2.97 (0.36)	85	3.04 (0.35)	−1.44
T4 ObAtt	408	3.00 (0.40)	1.68–4.24	196	3.09 (0.43)	212	2.92 (0.34)	1.90	335	3.02 (0.38)	73	2.94 (0.46)	1.58
T1 SocAnx	528	4.97 (3.43)	0–18	258	5.45 (3.83)	270	4.51 (2.93)	−4.97^***^	432	5.00 (3.43)	96	4.85 (3.43)	0.37
T2 SocAnx	497	4.70 (3.45)	0–18	238	5.48 (3.66)	259	3.99 (3.08)	−4.94^***^	410	4.68 (3.48)	87	4.78 (3.32)	−0.24
T3 SocAnx	478	5.06 (3.51)	0–16	229	6.28 (3.71)	249	3.94 (2.89)	−3.49^**^	392	4.95 (3.43)	86	5.57 (3.84)	−1.49
T4 SocAnx	437	5.68 (3.95)	0–18	211	7.28 (4.04)	226	4.19 (3.23)	−3.49^**^	356	5.66 (3.94)	81	5.80 (4.01)	−0.30
T1 Depress	528	4.60 (4.54)	0–26	258	4.69 (4.86)	270	4.50 (4.23)	−3.06^**^	432	4.59 (4.59)	96	4.62 (4.37)	−0.04
T2 Depress	498	3.85 (4.50)	0–23	239	4.36 (4.91)	259	3.36 (4.03)	−3.03^**^	411	3.81 (4.34)	87	4.05 (5.21)	−0.45
T3 Depress	478	4.26 (5.10)	0–26	229	5.57 (5.85)	249	3.05 (3.95)	−4.58^***^	392	4.03 (4.84)	86	5.28 (6.07)	−2.06^*^
T4 Depress	436	5.65 (6.62)	0–26	211	7.94 (7.07)	225	3.51 (4.33)	−4.54^***^	355	5.48 (6.03)	81	6.41 (6.98)	−1.21
T1 Life Sat	528	5.65 (1.00)	1.67–7	258	5.66 (1.06)	270	5.65 (0.93)	−0.10	432	5.69 (0.97)	96	5.50 (1.08)	1.71
T2 Life Sat	498	5.62 (1.08)	1.56–7	239	5.60 (1.12)	259	5.63 (1.05)	0.28	411	5.68 (1.05)	87	5.35 (1.20)	2.61^**^
T3 Life Sat	478	5.49 (1.11)	1.33–7	229	5.36 (1.19)	249	5.60 (1.01)	2.34^*^	392	5.56 (1.07)	86	5.16 (1.20)	3.06^**^
T4 Life Sat	435	5.30 (1.11)	1–7	210	5.03 (1.17)	225	5.55 (0.99)	−4.98^***^	354	5.35 (1.09)	81	5.06 (1.17)	2.16^*^
T1 Eat Path	525	5.05 (5.18)	0–48	257	5.23 (6.07)	268	4.87 (4.16)	−7.73^***^	429	4.94 (5.05)	96	5.51 (5.74)	−0.97
T2 Eat Path	498	4.49 (5.03)	0–53	239	4.67 (5.70)	259	4.31 (4.32)	−7.66^***^	411	4.54 (5.22)	87	4.25 (4.02)	0.48
T3 Eat Path	477	4.23 (4.95)	0–39	229	4.94 (5.75)	248	3.57 (3.97)	−8.86^***^	391	4.11 (4.96)	86	4.77 (4.91)	−1.11
T4 Eat Path	436	5.18 (7.29)	0–52	211	6.82 (9.01)	225	3.65 (4.71)	−8.79^***^	355	4.97 (7.09)	81	6.12 (8.07)	−1.29

*SubAtt* subjective attractiveness rating, *ObAtt* objective attractiveness rating, *SocAnx* social anxiety symptoms, *Depress* depressive symptoms, *Life Sat* life satisfaction, *Eat path* eating pathology symptoms, *T1* time 1, *T2* time 2, *T3* time 3, *T4* time 4

***p < 0.001, **p <0.01, *p < 0.05

**Table 2:** Pearson’s Bivariate Correlations between Study Variables

Variable name	1	2	3	4	5	6	7	8	9	10	11	12	13	14	15	16	17	18	19	20	21	22	23	24	25
1. T1 SubAtt	--																								
2. T1 ObAtt	0.21^**^	--																							
3. T1 SocAnx	−0.29^**^	−0.05	--																						
4. T1 Depress	−0.35^**^	−0.10^*^	0.59^**^	--																					
5. T1 LifeSat	0.42^**^	0.06	−0.44^**^	−0.59^**^	--																				
6. T1 EatPath	−0.30^**^	−0.09^*^	0.36^**^	0.39^**^	−0.36	--																			
7. T2SubAtt	0.43^**^	0.16^**^	−0.21^**^	−0.29^**^	0.31^**^	−0.22^**^	--																		
8. T2ObAtt	0.29^**^	0.70^**^	−0.07	−0.15^**^	0.11^*^	−0.11^*^	0.24^**^	--																	
9. T2SocAnx	−0.25^**^	−0.06	0.60^**^	0.44^**^	−0.34^**^	0.31^**^	−0.26^**^	−0.05	--																
10. T2Depress	−0.28^**^	−0.05	0.43^**^	0.53^**^	−0.46^**^	0.27^**^	−0.32^**^	−0.09^*^	0.59^**^	--															
11. T2LifeSat	0.28^**^	0.05	−0.31^**^	−0.40^**^	0.56^**^	−0.20^**^	0.36^**^	0.10^*^	−0.39^**^	−0.66^**^	--														
12. T2 EatPath	−0.23^**^	−0.04	0.31^**^	0.29^**^	−0.27^**^	0.55^**^	−0.26^**^	−0.04	0.43^**^	0.42^**^	−0.27^**^	--													
13. T3SubAtt	0.39^**^	0.09^*^	−0.24^**^	−0.30^**^	0.33^**^	−0.25^**^	0.47^**^	0.19^**^	−0.24^**^	−0.31^**^	0.34^**^	−0.21^**^	--												
14. T3ObAtt	0.26^**^	0.62^**^	−0.11^*^	−0.12^**^	0.08	−0.07	0.18^**^	0.72^**^	−0.09^*^	−0.06	0.06	−0.08	0.19^**^	--											
15. T3SocAnx	−0.14^**^	0.01	0.44^**^	0.30^**^	−0.24^**^	0.17^**^	−0.15^**^	−0.05	0.57^**^	0.38^**^	−0.24^**^	0.17^**^	−0.31^**^	−0.01	--										
16. T3Depress	−0.18^**^	0.02	0.31^**^	0.38^**^	−0.28^**^	0.21^**^	−0.13^**^	−0.04	0.41^**^	0.51^**^	−0.35^**^	0.18^**^	−0.33^**^	0.01	0.60^**^	--									
17. T3LifeSat	0.23^**^	0.03	−0.25^**^	−0.33^**^	0.44^**^	−0.18^**^	0.24^**^	0.09^*^	−0.34^**^	−0.47^**^	0.59^**^	−0.23^**^	0.42^**^	0.03	−0.46^**^	−0.70^**^	--								
18. T3 EatPath	−0.18^**^	0.03	0.26^**^	0.27^**^	−0.21^**^	0.37^**^	−0.13^**^	0.01	0.26^**^	0.20^**^	−0.14^**^	0.32^**^	−0.30^**^	0.01	0.40^**^	0.44^**^	−0.31^**^	--							
19. T4SubAtt	0.32^**^	0.10^*^	−0.23^**^	−0.25^**^	0.29^**^	−0.16^**^	0.42^**^	0.16^**^	−0.29^**^	−0.28^**^	0.31^**^	−0.21^**^	0.59^**^	0.11^*^	−0.29^**^	−0.32^**^	0.42^**^	−0.23^**^	--						
20. T4ObAtt	0.29^**^	0.56^**^	−0.10^*^	−0.11^*^	0.09	−0.05	0.20^**^	0.55^**^	−0.11^*^	0.11	0.13^**^	−0.04	0.22^**^	0.64^**^	−0.03	−0.05	0.08	0.03	0.14^**^	--					
21. T4SocAnx	−0.12^*^	−0.03	0.39^**^	0.29^**^	−0.20^**^	0.14^**^	−0.16^**^	−0.06	0.45^**^	0.25^**^	−0.16^**^	0.11^*^	−0.29^**^	−0.05	0.66^**^	0.46^**^	−0.38^**^	0.28^**^	−0.46^**^	−0.05	--				
22. T4Depress	−0.09	0.02	0.27^**^	0.28^**^	−0.25^**^	0.09	−0.12^*^	−0.02	0.31^**^	0.38^**^	−0.31^**^	0.11^*^	−0.23^**^	0.05	0.45^**^	0.56^**^	−0.47^**^	0.28^**^	−0.43^**^	0.07	−0.69^**^	--			
23. T4LifeSat	0.19^**^	0.05	−0.21^**^	−0.27^**^	0.36^**^	−0.08	0.26^**^	0.08	−0.25^**^	−0.35^**^	0.47^**^	−0.09	0.38^**^	0.06	−0.36^**^	−0.45^**^	0.62^**^	−0.23^**^	0.49^**^	0.07	−0.52^**^	−0.69^**^	--		
24. T4 EatPath	−0.19^**^	0.01	0.24^**^	0.24^**^	−0.23^**^	0.25^**^	−0.17^**^	−0.05	0.27^**^	0.25^**^	−0.21^**^	0.24^**^	−0.25^**^	0.01	0.30^**^	0.36^**^	−0.34^**^	0.36^**^	−0.41^**^	0.01	0.40^**^	0.53^**^	−0.41^**^	--	
25. Sex^a^	0.08	0.21^**^	0.14^*^	0.02	0.01	0.04	0.07	0.16^**^	0.22^**^	0.11^*^	−0.01	0.04	0.04	0.14^*^	0.33^**^	0.25^**^	−0.11^*^	0.14^*^	−0.09	0.22^**^	0.39^**^	0.36^**^	−0.23^**^	0.22^**^	--
26. Ethnicity^a^	0.04	0.03	−0.02	0.00	−0.07	0.04	−0.00	0.00	0.01	0.02	−0.12^*^	−0.02	−0.08	0.07	0.07	0.09^*^	−0.14^*^	0.05	−0.06	−0.08	0.02	0.06	−0.10^*^	0.06	0.00

*SubAtt* subjective attractiveness rating, *ObAtt* objective attractiveness rating, *SocAnx* social anxiety symptoms, *Depress* depressive symptoms, *LifeSat* life satisfaction, *EatPath* eating pathology symptoms, *T1* time 1, *T2* time 2, *T3* time 3, *T4* time 4

^**^p < 0.001, ^*^p < 0.05

^a^refers to point-biserial correlations

### Confirmatory Factor Analyses and Invariance Testing

The CFA of the latent internalizing factor provided an excellent fit to the data *(x*^*2*^ (*N* = 528, *df* = 30*)* = 38.57 (*p* = 0.136), CFI = 0.995, RMSEA = 0.023) with all factor loadings being positive, significant, and exceeding 0.50. The sex invariance testing revealed that all three models provided an excellent fit to the data (CFIs > 0.99 and RMSEAs < 0.029) and that the latent internalizing factor was fully invariant (i.e., △CFI < 0.01 for all model comparisons) across sex. Similarly, the ethnic group invariance testing revealed that all three models provided an excellent fit to the data (CFIs > 0.98 and RMSEAs < 0.040) and that the latent internalizing factor was fully invariant (i.e., △CFI < 0.01 for all model comparisons) across the White and non-White ethnic groups.

### Autoregressive Cross-lagged Panel Models

#### Prospective associations between subjective and objective attractiveness and internalizing symptoms

**Fig. 1:**
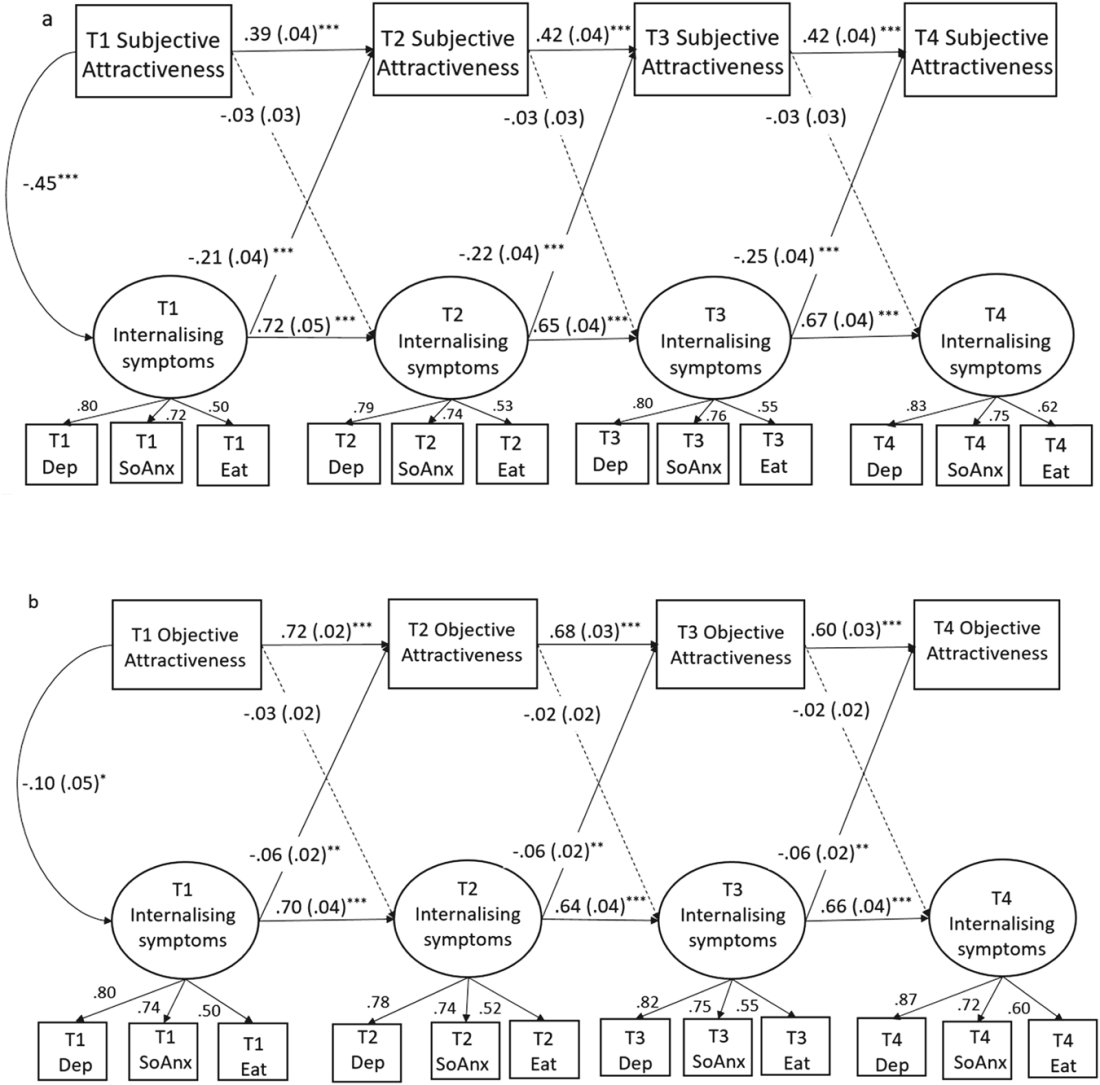
CLPMs between subjective (1a) and objective (1b) attractiveness and internalizing symptoms. Rectangles indicate observed variables, ellipses indicate latent factors. Paths represent standardized beta coefficients with standard errors in parentheses. Dashed lines represent paths that were estimated but were non-significant. Variable residuals were also correlated within waves at T2, T3, and T4 but arrows excluded here to aid interpretability. Dep depressive symptoms, SoAnx social anxiety symptoms. Eat eating pathology symptoms. ^*^p < 0.05, ^**^p < 0.01, ^***^p < 0.001

The model examining the bidirectional associations between subjective attractiveness and internalizing symptoms provided an acceptable fit to the data (*x*^*2*^ *(N* = 528, *df* = 98*)* = 236.78 (*p* < 0.001), CFI = 0.95, RMSEA = 0.052.). As shown in Fig. 1a, all items loaded well onto their designated factor (=> 0.50) and all autoregressive paths were positive and significant. The concurrent correlations between the two constructs (T1) and their residuals (T2-T4) were all negative (*r* = −0.23 to −0.51) and significant (*p’*s < 0.001). The paths from internalizing symptoms to subjective attractiveness one year later were significant across all time points but there was no evidence that changes in subjective attractiveness predicted later changes in internalizing symptoms.

The model examining the bidirectional associations between objective attractiveness and internalizing symptoms provided a good fit to the data (*x*^*2*^ (*N* = 520, *df* = 98) = 205.70 (*p* < 0.001), CFI = 0.96, RMSEA = 0.049). As shown in Fig. 1b, there were moderate to high levels of stability in objective attractiveness and internalizing symptoms across measurement occasions. There was a small significant concurrent correlation between objective attractiveness and internalizing symptoms at T1 (*r* = −0.10, *p* = 0.046) and their residual at T3 (*r* = −0.13, *p* = 0.041) but not at T2 (*r* = −0.01, *p* = 0.828) or T4 (*r* = −0.06, *p* = 0.313). The cross-lagged paths from internalizing symptoms to objective attractiveness were negative and significant across all waves, however, the effect sizes were small (*β*’s = −0.06). There were no significant associations from objective attractiveness to internalizing symptoms at any time point.

#### Prospective associations between objective and subjective attractiveness and life satisfaction

**Fig. 2:**
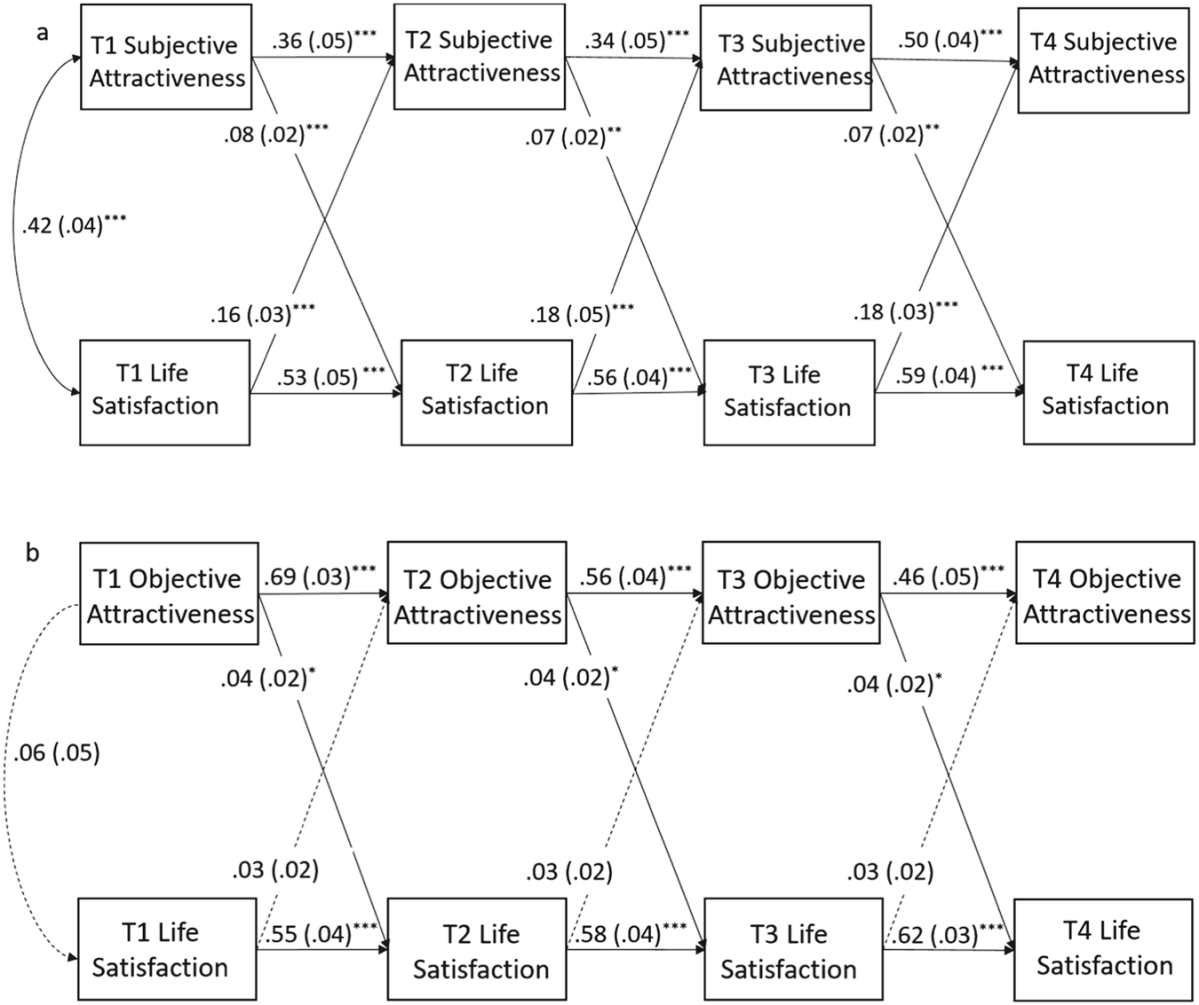
CLPMs between subjective (2a) and objective (2b) attractiveness and life satisfaction. *Note*. Rectangles indicate observed variables. Paths represent standardized beta coefficients with standard errors in parentheses. Dashed lines represent paths that were estimated but were non-significant. Variable residuals were also correlated within waves at T2, T3, and T4 but arrows excluded here to aid interpretability. ^*^p < 0.05, ^**^p < 0.01, ^***^p < 0.001

The model evaluating the bidirectional associations between subjective attractiveness and life satisfaction produced an acceptable fit to the data (*x*^*2*^ *(N* = 528, *df* = 21*)* = 63.37 (*p* < 0.001), CFI = 0.96, RMSEA = 0.062). Life satisfaction had moderate stability across waves and the T1 correlation between subjective attractiveness and life satisfaction was positive and significant (*r* = 0.42, *p* < 0.001) as were the concurrent residual correlations at T2 (*r* = 0.25, *p* < 0.001), T3 (*r* = 0.30, *p* < 0.001), and T4 (*r* = 0.28, *p* < 0.001). As shown in Fig. 2a, there was evidence of significant bidirectional associations between subjective attractiveness and life satisfaction across all time points, with changes in life satisfaction more strongly predicting changes in subjective attractiveness than the opposite association.

The model assessing the bidirectional associations between objective attractiveness and life satisfaction (see Fig. 2b) provided an adequate fit to the data (*x*^*2*^ *(N* = 528, *df* = 21*)* = 72.09 (*p* < 0.000), CFI = 0.96, RMSEA = 0.070). The within time correlations between the variables (T1) and their residuals (T2-T4) were all non-significant (*p’s* all > 0.172). The cross-lagged paths from objective attractiveness to life satisfaction were positive and significant. There were no significant associations in the other direction, although the effect size was similar.

### Moderating Analyses

The results of the moderating analyses revealed that neither sex (*p’s* > 0.574) nor ethnicity (*p’s* > 0.183) moderated any of the paths between objective attractiveness and internalizing symptoms. Similarly, sex and ethnicity did not moderate any of the paths between subjective attractiveness and internalizing symptoms (*p’s* > 0.376 and > 0.155 respectively). There was also no significant moderating effect of sex (*p’s* > 0.529) or ethnicity (*p’s* > 0.217) on the associations between subjective attractiveness and life satisfaction, or between objective attractiveness and life satisfaction (*p’s* > 0.278 and > 0.382 respectively). Thus, it was concluded that all of the associations tested in the primary analyses did not differ by sex or ethnic group.

## Discussion

There is a lack of current longitudinal research examining the bidirectional associations between attractiveness and mental health outcomes in adolescent samples. This is surprising considering the rise of social media over the past twenty years and adolescents’ corresponding greater exposure to beauty ideals and attractive people in an increasingly appearance-focused digital climate. The current study aimed to address this gap by examining the prospective bidirectional associations between changes in adolescents’ objective and subjective attractiveness and changes in life satisfaction and internalizing symptoms over four time points spanning early to mid-adolescence. Consistent with predictions, the study produced two main findings. First, changes in mental health over time were more strongly and consistently associated with changes in subjective attractiveness than objective attractiveness. Second, when there was evidence of prospective associations between either form of attractiveness and mental health outcomes, the associations appeared to be driven more by changes in mental health than by changes in either form of attractiveness. These findings were consistent across sex and ethnicity.

In terms of theory, social expectancy theories such as SCT (Berger et al., 1977) predict that greater physical attractiveness leads to better mental health, however, the current results provide only limited support for these predictions. While there were small, positive, prospective associations from both subjective and objective measures of attractiveness to life satisfaction, there was no evidence that increases in subjective or objective rated attractiveness predicted fewer internalizing symptoms. These findings support cross-sectional research showing more consistent evidence that objectively rated facial attractiveness is associated with life satisfaction (e.g., Diener et al., 1995), but less consistent evidence for significant associations with internalizing problems (e.g., McGovern et al., 1996).

Interestingly, the results more reliably indicated that increases in internalizing symptoms over time predicted significant decreases in both objectively and subjectively rated attractiveness, and that increases in life satisfaction predicted increases in subjective attractiveness, although the same relationship was not shown with objective attractiveness. Hence, it appears that those experiencing higher life satisfaction and lower internalizing symptoms perceive themselves, and are perceived by others, to be more attractive relative to those with lower life satisfaction and higher internalizing symptoms, a finding that cannot be explained by SCT or the attractiveness halo effect.

### Objective Attractiveness Internalizing Symptoms, and Life Satisfaction

Although the effect sizes were small, the objective attractiveness findings are consistent with a small body of research that has found that individuals high in anxiety, social anxiety, self-reported shyness, or disordered eating were rated by unfamiliar others as less attractive than healthy controls (Blöte et al., 2015; Jansen et al., 2006; Pilkonis, 1977; Reis et al., 1980) suggesting there are subtle, visible manifestations or characteristics of internalizing symptoms that may allow others to detect an individual’s internal distress. Such an interpretation is consistent with evolutionary perspectives of physical health, which predict that individuals with the highest fitness value (underlying health status), are perceived as the most attractive (Kniffin & Wilson, 2004). For example, Kramer and Ward (2010) demonstrated that physically healthy individuals can be accurately discriminated from unhealthy individuals simply by viewing a static image of a composite face. Although this finding was not replicated for mental health, images of those higher in psychological wellbeing were consistently rated as more attractive. Hence, consistent with the current results, it appears that a person’s internal mental state influences others’ ratings of their attractiveness, but the mechanism or process through which this occurs remains unclear.

One possible mechanism is that those with higher self-esteem at one time point are also less likely to have internalizing problems (Garcia-Reid et al., 2013), and in turn, their positive sense of self may cause them to present themselves (confident stance and facial expression) in ways that are viewed as more attractive by others. Other possible mechanisms may also include the tendency of individuals experiencing internalizing symptoms to spend less time on their physical appearance and self-care (e.g., poor hygiene; DiMatteo et al., 2000), hence appearing less attractive to others. Further, the current study used full body images for attractiveness ratings, thus those with symptoms of disordered eating and depression may have been rated as less attractive by others because the symptoms of some eating disorders (e.g., anorexia nervosa, binge eating) and depression can be physically manifested (e.g., low or high body weight).

Similarly, those experiencing social anxiety may outwardly exhibit symptoms such as a hunched or tense body posture, nervous facial expression, and/or lack of eye contact with the camera (Miers et al., 2013). Finally, although life satisfaction is not typically associated with physically observable symptoms it is possible that being happier and more satisfied with life results in subtle increases in facial animation and positive expression, and those more satisfied with their lives are are also more likely to engage in exercise and other health promoting behaviors (Grant et al., 2009), thus appearing more attractive. However, similar findings have been reported in studies using only head shots to rate facial attractiveness (Kramer & Ward, 2010), and when appearance enhancers such as makeup, hair, and clothing are removed (Little & Perrett, 2007), suggesting that factors other than self-care, grooming, and body weight may be involved, and that facial expression and features alone may be particularly important. Additional qualitative research with objective raters might provide more insight into the mechanisms and visual cues that people use to discriminate between individuals of varying mental health status, and how these cues in turn, influence their ratings of the target’s attractiveness.

### Subjective Attractiveness, Internalizing Symptoms, and Life Satisfaction

In terms of subjective attractiveness, the current results align with previous longitudinal evidence showing that depressive symptoms significantly predicted later decreases in subjective attractiveness in children and adolescents, while finding no evidence for the inverse association (Cole et al., 1998). Our results extend these findings to the general variance that is common between different forms of internalizing distress. These findings support clinical cognitive theories of internalizing disorders and depression in particular, whereby self-devaluative thinking and negative biases are involved in the maintenance of internalizing disorders (Dent & Teasdale, 1988). However, given the tendency of healthy individuals to overestimate their own attractiveness (Greitemeyer, 2020), it may be that rather than a negative cognitive biases lowering self-perceived attractiveness, it could be that current results reflect the lack of a positive bias among those with higher internalizing symptoms (Jansen et al., 2006). It is possible that self-esteem also plays a role in the predictive association from internalizing difficulties to subjective attractiveness. Consistent with the scar model of depression and self-esteem (Coyne et al., 1998), internalizing symptoms may erode self-esteem over time, which in turn may lead to lower self-perceptions of attractiveness. Therefore, it is recommended that future research explore additional potential moderators and mediators of the associations found in the current research.

In contrast to the current unidirectional results found for changes in internalizing symptoms preceding changes in subjective attractiveness, there were bidirectional associations found between life satisfaction and subjective attractiveness. While these significant associations replicate well-established correlational and previous unidirectional findings (Diener et al., 1995; Hamermesh & Abrevaya, 2013; Skałacka & Pajestka, 2021; Talbot, 2012), this is the first study to test this association bidirectionally. It is important to note however, that the paths leading from life satisfaction to subjective attractiveness were twice the strength of those leading from subjective attractiveness to life satisfaction. This suggests that, like internalizing symptoms, change in life satisfaction is a stronger predictor of change in subjective attractiveness than the opposite association, although more research is needed to replicate these findings.

### Group differences

Overall, the lack of sex difference findings were inconsistent with early predictions of objectification (Fredrickson & Roberts, 1997) and sociocultural theory (Thompson et al., 1999), which predict that the associations between attractiveness and mental health outcomes may be stronger for girls/women due to early socialisation experiences emphasizing the importance of being physically attractive and increased pressure to attain unrealistic beauty ideals (Moradi & Huang, 2008). Instead it is possible that the current findings reflect the increasing sexual objectification of boys/men in recent decades, along with greater societal emphasis on male body ideals (e.g., drive for masculinity), and the increase in the internalization of these unattainable beauty standards among boys and young men (Parent & Moradi, 2011; Wiseman & Moradi, 2010). As such, the lack of sex differences found in the current study suggests that these theories, and their predicted associations with mental health, are equally relevant to boys and men. This is supported by the recent findings that there are no sex differences in the amount of time men and women invest in enhancing their attractiveness (Kowal & Sorokowski, 2022), and the increased rates of body disturbance, steroid use, and eating disorders in boys and young men (Nagata et al., 2020).

The results also revealed high consistency in attractiveness ratings between raters of different ethnic backgrounds along with no ethnic differences in the prospective bidirectional associations between attractiveness, internalizing symptoms, and life satisfaction. Before interpreting this result, it needs to be repeated that we did not have a large sample of non-White participants and therefore had to combine very disparate ethnic groups under a general “non-White” category, potentially weakening possible specific ethnicity effects. Nonetheless, the main finding as it stands is consistent with suggestions that the globalization of Western media and advertising through digital technology has resulted in universally endorsed attractiveness standards (Isa & Kramer, 2003). The results also align with previous studies that have found a high level of agreement among raters from different cultural backgrounds (e.g., Kočnar et al., 2019) again suggesting that Western beauty standards have superseded any previous local differences reported in early research (Yan & Bissell, 2014). Finally, this study extends the existing literature by demonstrating that not only are judgements of what is and is not attractive becoming more universal but also how self and other perceived attractiveness relates to changing levels of life satisfaction and internalizing symptoms.

### Implications

The current results challenge existing social expectancy theories such as SCT (Berger et al., 1977) and the attractiveness halo effect (Dion et al., 1972), as well as the tripartite socio-cultural model (e.g., Thompson et al., 1999) all of which predict a positive unidirectional association from attractiveness to mental health. Instead, the direction of the association is more consistent with clinical cognitive theories and the negative biases associated with internalizing disorders (Dent & Teasdale, 1988), specifically that those experiencing more internalizing symptoms and lower life satisfaction are more likely to perceive themselves and be perceived by others as less attractive.

Yet it is probable that these theories are not mutually exclusive. For example, integrating both clinical cognitive and socio-cultural models in relation to subjective attractiveness, it is possible that those with internalizing problems may judge their appearance more harshly when comparing their physical appearance to societal beauty ideals due to the negative processing biases and self-devaluative thinking associated with these disorders (Orchard & Reynolds, 2018). It is also possible that those experiencing symptoms of depression, social anxiety, and eating disorders internalize unrealistic beauty standards to a greater extent than their more adjusted counterparts, and thus experience larger discrepancies between the actual and ideal self, in turn leading to greater decreases in self-perceived attractiveness (Stice & Whitenton, 2002). Further exploration of the integration of these different theories is warranted, however, there is some preliminary evidence in the broader body image literature that depressive symptoms are associated with greater internalization of the thin-ideal and serve as a precursor to body image concerns (Rodgers et al., 2014).

Similarly, social expectancy theories cannot fully account for the associations found between objective attractiveness, life satisfaction and internalizing symptoms. Consistent with the halo effect and SCT, there were small positive effects for objective attractiveness on life satisfaction which suggests that the social advantages afforded to attractive people may lead to small increases in overall life satisfaction. However, the same effect does not appear to generalize to a reduction in internalizing symptoms and the association between life satisfaction and objective ratings of attractiveness was much larger in the opposite direction. Thus, as suggested above, other theories such as evolutionary theory (Kniffin & Wilson, 2004) should also be explored to complement and extend upon social expectancy theories of attractiveness. Overall, the current research findings highlight the importance of looking beyond our silos and incorporating a broad range of theories across different fields of psychology to provide a more holistic understanding of social phenomenon.

The results from the current study may also help inform prevention and intervention efforts targeting those using unhealthy and harmful methods to change their appearance due to low self-perceived attractiveness. Within the body image literature, many existing initiatives and treatments designed to address concerns about appearance promote body diversity and acceptance in efforts prevent subsequent mental health problems (Margolis & Orsillo, 2016). However, the current findings demonstrating that changes in internalizing symptoms preceded decreases in self-perceived attractiveness suggest that additional strategies such as teaching adolescents to identify internalizing symptoms and to challenge existing stereotypes surrounding the social and psychological benefits of being attractive (i.e., the halo effect) may also be valuable in reducing negative self-perceptions of attractiveness.

Dispelling well-established stereotypes is notoriously difficult, therefore researchers, parents, and educators need to find creative and effective ways of educating adolescents that the stereotypes around beauty are largely perception-based and that attractiveness should not be critical to self-worth. Considering its popularity among young people, social media may provide an effective means by which to disseminate anti-stereotypical information to adolescents. While social media is often cited as a contributor to appearance concerns (e.g., Fardouly et al., 2020), existing Facebook and Instagram campaigns promoting the acceptance of diverse body sizes and physical appearances have had some success in challenging societal messaging around beauty ideals (Cohen et al., 2021). Therefore, extending such campaigns to include popular and attractive influencer’s sharing their experiences of mental health difficulties may assist in further challenging common beauty stereotypes linking attractiveness to happiness.

The current results may also have implications for addressing appearance-based peer rejection and victimization. Existing research shows that physical appearance is the most common reason that adolescents are bullied or rejected by peers (Puhl et al., 2016). Perhaps relatedly, there has also been an increase in the number of adolescents undergoing cosmetic procedures in recent years (American Society of Plastic Surgeons, 2021) with the most common reason given by adolescents for having the procedure being to fit in with, and look more acceptable to, their peers (American Society of Plastic Surgeons, 2021). However, the current results, along with research showing that adolescents experiencing internalizing symptoms are at greater risk of peer victimization and rejection (Christina et al., 2021), indicate that rather than trying to change one’s appearance, routine mental health screening and early intervention may assist in both increasing subjective and objective attractiveness, and reducing the risk of peer rejection and victimization.

### Limitations

The results of the current study should be interpreted in light of several limitations. First, although the data collected was longitudinal, true causality cannot be determined with the current design and analyses. Therefore, future research should further explore the associations examined in the current study employing experimental methods. Second, the present study used a latent variable to measure internalizing symptoms because the focus of interest was on the common variance shared between different forms of internalizing disorder (Kotov et al., 2017). As a result, the relationships between objective and subjective attractiveness and the characteristics of anxiety, depression and eating pathology that are uniquely associated with each of these problems, could not be examined. Now that these preliminary bidirectional associations have been established with the common variance across internalizing symptoms, future research could partial out this common variance to investigate unique variance associated with individual disorders to identify any disorder-specific nuances among the associations tested. Third, the convenience sample obtained in the current study lacked cultural diversity, as all participants lived in the western country of Australia, were predominantly white, and spoke English as a first language. Although comparisons across white and non-white ethnic groups was made, this rather rudimentary division fails to account for the diverse range of ethnicities within the non-white group. Therefore, while previous research (e.g., Yan & Bissell, 2014) and the current findings suggest that attractiveness standards are universal, there has been very limited research exploring the correlates of attractiveness across ethnic groups and further research better delineating a broader range of cultural groups is needed to substantiate the current results. Fourth, the raters within the present study were all adults and primarily white. It is possible that findings may vary with adolescent or more culturally diverse raters, and hence the study could be replicated using peer raters from multiple ethnic groups. However, minimizing this as a threat to the validity of the current findings, research shows that objective ratings of attractiveness are remarkably similar irrespective of raters’ age, culture, or sex (Langlois et al., 2000).

## Conclusion

Existing research examining the associations between objective and subjective attractiveness and a range of mental health outcomes in adolescents and adults has largely been cross-sectional and when longitudinal designs are employed, the main focus has been on the unidirectional association from attractiveness to mental health outcomes. The present study extended upon the existing literature by providing prospective directional evidence that changes in both positive (life satisfaction) and negative (internalizing symptoms) aspects of mental health over time are more strongly and consistently associated with changes in subjective attractiveness than objective attractiveness. This finding is important as over the course of adolescence there is a growing focus on physical appearance, along with a marked increase in the onset of a range of social emotional disorders. The results also demonstrated that changes in life satisfaction and internalizing symptoms predicted changes in subjective and objective attractiveness ratings with only limited evidence of the opposite association. These findings are largely inconsistent with the “physical attractiveness halo effect”, instead pointing to the need for new messaging aimed at challenging existing stereotypes around the psychological “benefits of beauty.” There is also a need to find effective ways of educating adolescents who are unhappy with their appearance, that making changes to improve their mental health, rather than focusing on their physical appearance, will have benefits not only for how they perceive themselves but also how they are perceived by others.

## Appendix

Tables 3–6

**Table 3:** Pearson’s Bivariate Correlations between Study Variables for Boys

Variable	1	2	3	4	5	6	7	8	9	10	11	12	13	14	15	16	17	18	19	20	21	22	23
1. T1 SubAtt	--																						
2. T1 ObAtt	0.19^**^	--																					
3. T1 SocAnx	−0.24^**^	−0.06	--																				
4. T1 Depress	−0.29^**^	−0.12^*^	0.49^**^	--																			
5. T1 LifeSat	0.41^**^	0.11	−0.46^**^	−0.51^**^	--																		
6. T1 EatPath	−0.22^**^	−0.22^*^	0.31^**^	0.30^**^	−0.23^**^	--																	
7. T2SubAtt	0.53^**^	0.13^*^	−0.20^**^	−0.24^**^	0.27^**^	−0.11	--																
8. T2ObAtt	0.32^**^	0.69^**^	−0.10	−0.20^**^	0.16^**^	−0.16^*^	0.26^**^	--															
9. T2SocAnx	−0.28^**^	−0.11	0.55^**^	0.36^**^	−0.30^**^	0.29^**^	−0.28^**^	−0.10	--														
10. T2Depress	−0.30^**^	−0.12	0.44^**^	0.53^**^	−0.43^**^	0.27^**^	−0.34^**^	−0.17^**^	0.56^**^	--													
11. T2LifeSat	0.36^**^	0.05	−0.36^**^	−0.35^**^	0.56^**^	−0.17^**^	0.35^**^	0.15^*^	−0.34^**^	−0.64^**^	--												
12. T2 EatPath	−0.13^**^	−0.19^**^	0.23^**^	0.18^**^	−0.13^**^	0.51^**^	−0.09^**^	−0.10	0.37^**^	0.36^**^	−0.17^**^	--											
13. T3SubAtt	0.45^**^	0.06	−0.23^**^	−0.26^**^	0.27^**^	−0.16^*^	0.48^**^	0.20^**^	−0.25^**^	−0.31^**^	0.41^**^	−0.18^**^	--										
14. T3ObAtt	0.34^**^	0.62^**^	−0.13^*^	−0.16^**^	0.16^*^	−0.14^*^	0.19^**^	0.73^**^	−0.10^*^	−0.16^*^	0.13^*^	−0.19^**^	0.20^**^	--									
15. T3SocAnx	−0.20^**^	−0.04	0.33^**^	0.18^**^	−0.17^**^	0.13^*^	−0.18^**^	−0.14^*^	0.54^**^	0.27^**^	−0.21^**^	0.17^**^	−0.28^**^	−0.12^*^	--								
16. T3Depress	−0.25^**^	0.12	0.23^*^	0.34^**^	−0.21^**^	0.22^**^	−0.18^**^	−0.25^**^	0.37^**^	0.50^**^	−0.34^**^	0.19^**^	−0.31^**^	−0.17^**^	0.47^**^	--							
17. T3LifeSat	0.31^**^	0.12	−0.19^*^	−0.26^**^	0.38^**^	−0.15^*^	0.23^**^	0.21^**^	−0.31^**^	−0.43^**^	0.58^**^	−0.16^**^	−0.20^**^	−0.07	0.32^**^	0.63^**^	--						
18. T3EatPath	−0.12^*^	−0.06	0.11	0.16^**^	−0.13^*^	0.36^**^	−0.05	−0.08	0.18^**^	0.15^*^	−0.12^*^	0.34^**^	0.41^**^	0.16^*^	−0.35^**^	−0.39^**^	−0.21^**^	--					
19. T4SubAtt	0.39^**^	0.15^*^	−0.23^**^	−0.22^**^	0.29^**^	−0.07	0.42^**^	0.19^**^	−0.30^**^	−0.26^**^	0.34^**^	−0.14^*^	0.57^**^	0.18^**^	−0.28^**^	−0.32^**^	0.42^**^	−0.12^*^	--				
20. T4ObAtt	0.31^**^	0.45^**^	−0.13	−0.13	0.08	−0.08	0.22^**^	0.47^**^	−0.15^*^	0.13^*^	0.13^*^	−0.09	0.26^**^	0.63^**^	−0.13	−0.11	0.15^*^	0.00	0.23^**^	--			
21. T4SocAnx	−0.17^*^	−0.03	0.24^**^	0.24^**^	−0.13^*^	−0.06	−0.13	−0.09	0.37^**^	0.17^**^	−0.18^*^	0.09	−0.36^**^	−0.08	0.60^**^	0.39^**^	−0.34^**^	0.23^**^	−0.49^**^	−0.15^*^	--		
22. T4Depress	−0.19^**^	0.06	0.14^*^	0.31^**^	−0.24^**^	0.10	−0.16^*^	−0.05	0.27^**^	0.38^**^	−0.34^**^	0.09	−0.32^**^	−0.07	0.33^**^	0.59^**^	−0.48^**^	0.24^**^	−0.40^**^	−0.01	−0.52^**^	--	
23. T4LifeSat	0.29^**^	0.08	−0.15^*^	−0.28^**^	0.36^**^	−0.05	0.22^**^	0.08	−0.23^**^	−0.36^**^	0.48^**^	−0.07	−0.37^**^	0.08	−0.27^**^	0.45^**^	0.63^**^	−0.12^*^	0.38^**^	0.14^*^	−0.46^**^	−0.65^**^	--
24. T4EatPath	−0.22^**^	−0.03	0.08	0.23^**^	−0.19^**^	0.17^*^	−0.07	−0.02	0.20^**^	0.29^**^	−0.26^**^	0.15^**^	0.29^**^	−0.01	0.21^**^	−0.41^**^	−0.35^**^	0.43^**^	−0.30^**^	0.01	0.30^**^	0.39^**^	−0.28^**^

*SubAtt* subjective attractiveness rating, *ObAtt* objective attractiveness rating, *SocAnx* social anxiety symptoms, *Depress* depressive symptoms, *LifeSat* life satisfaction, *EatPath* eating pathology symptoms, *T1* time 1, *T2* time 2, *T3* time 3, *T4* time 4

^**^*p* < 0.001, ^*^*p* < 0.05

**Table 4:** Pearson’s Bivariate Correlations between Study Variables for Girls

Variable	1	2	3	4	5	6	7	8	9	10	11	12	13	14	15	16	17	18	19	20	21	22	23
1. T1SubAtt	--																						
2. T1ObAtt	0.20^**^	--																					
3. T1SocAx	−0.34^**^	−0.10	--																				
4. T1Dep	−0.41^**^	−0.10	0.66^**^	--																			
5. T1LifSat	0.44^**^	0.03	−0.45^**^	−0.66^**^	--																		
6. T1Eatpath	−0.37^**^	−0.04	0.39^**^	0.45^**^	−0.45^**^	--																	
7. T2SubAtt	0.35^**^	0.16^*^	−0.24^**^	−0.33^**^	0.34^**^	−0.30^**^	--																
8. T2ObAtt	0.24^**^	0.70^**^	−0.10	−0.11	0.06	−0.10	0.20^**^	--															
9. T2SocAx	−0.28^**^	−0.11	0.62^**^	0.52^**^	−0.40^**^	0.32^**^	−0.29^**^	−0.08	--														
10. T2Dep	−0.29^**^	−0.04	0.42^**^	0.53^**^	−0.49^**^	0.25^**^	−0.33^**^	−0.06	0.61^**^	--													
11. T2LifSat	0.22^**^	0.06	−0.28^**^	−0.43^**^	0.56^**^	−0.22^**^	0.38^**^	0.05	−0.45^**^	−0.68^**^	--												
12. T2EatPath	−0.31^**^	0.04	0.35^**^	0.37^**^	−0.38^**^	0.58^**^	−0.38^**^	0.01	0.48^**^	0.46^**^	−0.35^**^	--											
13. T3SubAttM	0.33^**^	0.10	−0.23^**^	−0.34^**^	0.38^**^	−0.32^**^	0.46^**^	0.18^**^	−0.27^**^	−0.27^**^	0.27^**^	−0.23^**^	--										
14. T3ObAtt	0.19^**^	0.60^**^	−0.16^*^	−0.10	0.03	−0.04	0.15^*^	0.71^**^	−0.15^**^	−0.02	−0.01	−0.01	0.17^*^	--									
15. T3SocAx	−0.16^*^	−0.08	0.48^**^	0.40^**^	−0.31^**^	0.19^**^	−0.19^**^	−0.07	0.55^**^	0.43^**^	−0.28^**^	0.17^**^	−0.39^**^	−0.01	--								
16. T3Dep	−0.17^**^	0.01	0.31^**^	0.41^**^	−0.34^**^	0.19^**^	−0.13^*^	0.04	0.38^**^	0.50^**^	−0.39^**^	0.17^**^	−0.38^**^	0.06	0.62^**^	--							
17. T3LifSat	0.18^**^	0.00	−0.27^**^	−0.38^**^	0.49^**^	−0.20^**^	0.25^**^	0.01	−0.34^**^	−0.50^**^	0.60^**^	−0.27^**^	0.45^**^	−0.04	−0.52^**^	−0.74^**^	--						
18. T3Eatpath	−0.24^**^	0.05	0.31^**^	0.34^**^	−0.26^**^	0.37^**^	−0.19^**^	0.05	0.28^**^	0.21^**^	−0.16^*^	0.31^**^	−0.39^**^	0.03	0.41^**^	0.43^**^	−0.35^**^	--					
19. T4SubAtt	0.28^**^	0.09	−0.21^**^	−0.27^**^	0.29^**^	−0.21^**^	0.44^**^	0.16^*^	−0.27^**^	−0.28^**^	0.28^**^	−0.26^**^	0.62^**^	0.08	−0.29^**^	−0.31^**^	0.41^**^	−0.30^**^	--				
20. T4ObAtt	0.27^**^	0.60^**^	−0.15^*^	−0.11	0.12	−0.06	0.17^*^	0.58^**^	−0.18^*^	−0.14	0.13	−0.01	0.17^*^	0.62^**^	−0.11	−0.11	0.08	−0.01	0.13^*^	--			
21. T4SocAx	−0.17^*^	−0.18^**^	0.45^**^	0.36^**^	−0.27^**^	0.18^**^	−0.28^**^	−0.17^*^	0.43^**^	0.27^**^	−0.19^**^	0.12	−0.33^**^	−0.15^*^	0.61^**^	0.42^**^	−0.39^**^	0.25^**^	−0.44^**^	−0.16^*^	--		
22. T4Dep	−0.09	−0.05	0.28^**^	0.29^**^	−0.28^**^	0.07	−0.17^*^	−0.09	0.25^**^	0.36	−0.35^**^	0.13	−0.25^**^	0.02	0.40^**^	0.48^**^	−0.47^**^	0.26^**^	−0.45^**^	−0.02	0.61^**^	--	
23. T4LifSat	0.15^*^	0.12	−0.20^**^	−0.27^**^	0.38^**^	−0.09	0.35^**^	0.15^**^	−0.20^**^	−0.31^**^	0.49^**^	−0.10	0.43^**^	0.10	−0.33^**^	−0.40^**^	0.60^**^	−0.26^**^	0.57^**^	0.13	−0.49^**^	−0.70^**^	--
T4EatPath	−0.21^**^	−0.07	0.28^**^	0.25^**^	−0.26^**^	0.28^**^	−0.25^**^	−0.12	0.26^**^	0.22^**^	−0.21^**^	0.28^**^	−0.26^**^	−0.05	0.26^**^	0.30^**^	−0.32^**^	0.31^**^	−0.47^**^	−0.07	0.39^**^	0.54^**^	−0.44^**^

*SubAtt* subjective attractiveness rating, *ObAtt* objective attractiveness rating, *SocAnx* social anxiety symptoms, *Depress* depressive symptoms, *LifeSat* life satisfaction, *EatPath* eating pathology symptoms, *T1* time --, *T2* time 2, *T3* time 3, *T4* time 4

^**^*p* < 0.001, ^*^*p* < 0.05

**Table 5:** Pearson’s Bivariate Correlations between Study Variables for the White Ethnic Group

	1	2	3	4	5	6	7	8	9	10	11	12	13	14	15	16	17	18	19	20	21	22	23
1. T1SubAtt	--																						
2. T1ObAtt	0.14^**^	--																					
3. T1SocAx	−0.30^**^	−0.04	--																				
4. T1Dep	−0.38^**^	−0.10^*^	0.59^**^	--																			
5. T1LifSat	0.44^**^	0.02	−0.45^**^	−0.60^**^	--																		
6. T1Eatpath	−0.33^**^	−0.06	0.40^**^	0.40^**^	−0.36^**^	--																	
7. T2SubAtt	0.41^**^	0.13^**^	−0.21^**^	−0.30^**^	0.30^**^	−0.23^**^	--																
8. T2ObAtt	0.24^**^	0.66^**^	−0.05	−0.14^**^	0.07	−0.09	0.22^**^	--															
9. T2SocAx	−0.27^**^	−0.02	0.62^**^	0.45^**^	−0.37^**^	0.34^**^	−0.29^**^	−0.02	--														
10. T2Dep	−0.28^**^	−0.01	0.41^**^	0.51^**^	−0.44^**^	0.31^**^	−0.34^**^	−0.04	0.59^**^	--													
11. T2LifSat	0.23^**^	0.01	−0.30^**^	−0.38^**^	0.53^**^	−0.21^**^	0.39^**^	0.05	−0.39^**^	−0.66^**^	--												
12. T2EatPath	−0.25^**^	−0.03	0.34^**^	0.31^**^	−0.31^**^	0.57^**^	−0.29^**^	−0.03	0.46^**^	0.49^**^	−0.31^**^	--											
13. T3SubAttM	0.41^**^	0.07	−0.28^**^	−0.32^**^	0.30^**^	−0.24^**^	0.45^**^	0.18^**^	−0.27^**^	−0.34^**^	0.33^**^	−0.21^**^	--										
14. T3ObAtt	0.23^**^	0.60^**^	−0.11^*^	−0.13^**^	0.08	−0.07	0.16^**^	0.71^**^	−0.08	−0.04	0.05	−0.07	0.20^**^	--									
15. T3SocAx	−0.16^**^	0.04	0.41^**^	0.26^**^	−0.19^**^	0.16^**^	−0.15^**^	−0.00	0.57^**^	0.34^**^	−0.16^**^	0.20^**^	−0.33^**^	−0.01	--								
16. T3Dep	−0.17^**^	0.03	0.27^**^	0.33^**^	−0.21^**^	0.17^**^	−0.10^**^	−0.019	0.40^**^	0.49^**^	−0.31^**^	0.21^**^	−0.33^**^	0.01	0.57^**^	--							
17. T3LifSat	0.23^**^	−0.02	−0.25^**^	−0.30^**^	0.39^**^	−0.17^**^	0.23^**^	0.03	−0.33^**^	−0.46^**^	0.54^**^	−0.24^**^	0.41^**^	0.028	−0.43^**^	−0.69^**^	--						
18. T3Eatpath	−0.20^**^	0.01	0.28^**^	0.26^**^	−0.19^**^	0.38^**^	−0.13^**^	0.01	0.29^**^	0.21^**^	−0.12^*^	0.34^**^	−0.28^**^	−0.01	0.39^**^	0.42^**^	−0.28^**^	--					
19. T4SubAtt	0.33^**^	0.08	−0.28^**^	−0.27^**^	0.26^**^	−0.16^**^	0.42^**^	0.13^*^	−0.33^**^	−0.30^**^	0.28^**^	−0.22^**^	0.56^**^	0.10	−0.33^**^	−0.33^**^	0.39^**^	−0.23^**^	--				
20. T4ObAtt	0.28^**^	0.53^**^	−0.10	−0.15^**^	0.08	−0.06	0.18^**^	0.52^**^	−0.08	−0.10	0.10	−0.05	0.22^**^	0.64^**^	0.02	−0.03	0.03	0.01	0.15^**^	--			
21. T4SocAx	−0.14^*^	−0.02	0.36^**^	0.27^**^	−0.15^**^	0.09	−0.17^**^	−0.05	0.45^**^	0.21^**^	−0.10	0.12^*^	−0.31^**^	−0.06	0.64^**^	0.44^**^	−0.38^**^	0.25^**^	−0.52^**^	−0.054	--		
22. T4Dep	−0.10	−0.01	0.26^**^	0.26^**^	−0.21^**^	0.08	−0.12^*^	0.01	0.32^**^	0.34^**^	−0.25^**^	0.14^**^	−0.22^**^	0.03	0.42^**^	0.55^**^	−0.46^**^	0.23^**^	−0.44^**^	0.05	0.64^**^	--	
23. T4LifSat	0.19**	0.08	−0.24^**^	−0.29^**^	0.34^**^	−0.07	0.27^**^	0.07	−0.28^**^	−0.35^**^	0.45^**^	−0.11^*^	0.35^**^	0.08	−0.34^**^	−0.43^**^	0.60^**^	−0.20^**^	0.48^**^	0.06	−0.54^**^	−0.69^**^	--
24. T4EatPath	−0.18**	0.01	0.27^**^	0.25^**^	−0.22^**^	0.27^**^	−0.17^**^	−0.01	0.30^**^	0.24^**^	−0.17^**^	0.28^**^	−0.25^**^	−0.01	0.31^**^	0.35^**^	−0.31^**^	0.36^**^	−0.41^**^	0.00	0.41^**^	0.51^**^	−0.36^**^

*SubAtt* subjective attractiveness rating, *ObAtt* objective attractiveness rating, *SocAnx* social anxiety symptoms, *Depress* depressive symptoms, *LifeSat* life satisfaction, *EatPath* eating pathology symptoms, *T1* time --, *T2* time 2, *T3* time 3, *T4* time 4

^**^*p* < 0.001, ^*^*p* < 0.05

**Table 6:** Pearson’s Bivariate Correlations between Study Variables for the non-White Ethnic Group

	1	2	3	4	5	6	7	8	9	10	11	12	13	14	15	16	17	18	19	20	21	22	23
1. T1SubAtt	--																						
2. T1ObAtt	0.50^**^	--																					
3. T1SocAx	−0.20	−0.10	--																				
4. T1Dep	−0.21^*^	−0.12	0.60^**^	--																			
5. T1LifSat	0.34^**^	0.24^*^	−0.43^**^	−0.54^**^	--																		
6. T1Eatpath	−0.20	−0.22^*^	0.22^*^	0.34^**^	−0.35^**^	--																	
7. T2SubAtt	0.54^**^	0.29^**^	−0.22^*^	−0.18	0.38^**^	−0.20	--																
8. T2ObAtt	0.53^**^	0.82^**^	−0.17	−0.19	0.26^*^	−0.20	0.31^**^	--															
9. T2SocAx	−0.17	−0.24^*^	0.52^**^	0.42^**^	−0.22^*^	0.17	−0.13	−0.18	--														
10. T2Dep	−0.29^**^	−0.20	0.56^**^	0.64^**^	−0.51^**^	0.09	−0.25^*^	−0.28^**^	0.64^**^	--													
11. T2LifSat	0.24^*^	0.23^*^	−0.38^**^	−0.46^**^	0.67^**^	−0.15	0.26^*^	0.27^*^	−0.41^**^	−0.65^**^	--												
12. T2Eatpath	−0.12	−0.13	0.14	0.17	−0.13	0.48^**^	−0.05	−0.10	0.26^*^	0.12	−0.14	--											
13. T3SubAtt	0.28^**^	0.18	−0.05	−0.21	0.43^**^	−0.27^*^	0.55^**^	0.23^*^	−0.11	−0.19	0.33^**^	−0.21^*^	--										
14. T3ObAtt	0.41^**^	0.73^**^	−0.10	−0.09	0.15	−0.07	0.29^**^	0.80^**^	−0.14	−0.16	0.13	−0.11	0.15	--									
15. T3SocAx	−0.04	−0.15	0.58^**^	0.49^**^	−0.39^**^	0.22^*^	−0.18	−0.20	0.58^**^	0.54^**^	−0.47^**^	0.05	−0.20	−0.01	--								
16. T3Dep	−0.18	−0.04	0.48^**^	0.56^**^	−0.49^**^	0.32^**^	−0.25^*^	−0.12	0.44^**^	0.57^**^	−0.47^**^	0.08	−0.31^**^	0.01	0.67^**^	--							
17. T3LifSat	0.22^*^	0.22^*^	−0.28^**^	−0.43^**^	0.56^**^	−0.23^*^	0.26^*^	0.22^*^	−0.39^**^	−0.54^**^	0.71^**^	−0.21	0.47^**^	0.09	−0.52^**^	−0.70^**^	--						
18. T3Eatpath	−0.05	0.11	0.16	0.31^**^	−0.28^**^	0.33^**^	−0.08	0.01	0.10	0.15	−0.20	0.24^*^	−0.37^**^	0.12	0.42^**^	0.49^**^	−0.39^**^	--					
19. T4SubAtt	0.25^*^	0.16	0.02	−0.16	0.39^**^	−0.17	0.44^**^	0.26^*^	−0.11	−0.20	0.38^**^	−0.16	0.72^**^	0.15	−0.123	−0.27^*^	0.54^**^	−0.22^*^	--				
20. T4ObAtt	0.36^**^	0.70^**^	−0.11	0.04	0.11	0.00	0.29^*^	0.61^**^	−0.27^*^	−0.13	0.22	−0.00	0.17	0.67^**^	−0.18	−0.08	0.21	0.10	0.11	--			
21. T4SocAx	−0.05	−0.09	0.54^**^	0.39^**^	−0.38^**^	0.36^**^	−0.12	−0.13	0.45^**^	0.42^**^	−0.42^**^	0.02	−0.24^*^	−0.01	0.75^**^	0.54^**^	−0.43^**^	0.40^**^	−0.22	−0.04	--		
22. T4Dep	−0.02	0.11	0.32^**^	0.38^**^	−0.38^**^	0.12	−0.11	−0.11	0.27^*^	0.53^**^	−0.51^**^	−0.03	−0.29^*^	0.09	0.58^**^	0.56^**^	−0.51^**^	0.46^**^	−0.39^**^	0.14	0.64^**^	--	
23. T4LifSat	0.19	−0.02	−0.12	−0.21	0.44^**^	−0.11	0.21	0.11	−0.11	−0.34^**^	0.53^**^	0.01	0.45^**^	0.01	−0.41^**^	−0.47^**^	0.68^**^	−0.33^**^	0.51^**^	0.07	−0.44^**^	−0.70^**^	--
24. T4Eatpath	−0.20	0.01	0.14	0.18	−0.25^*^	0.18	−0.14	−0.17	0.14	0.31^**^	−0.34^**^	0.04	−0.23^*^	0.00	0.25^*^	0.39^**^	−0.42^**^	0.35^**^	−0.41^**^	0.04	0.37^**^	0.60^**^	−0.58^**^

*SubAtt* subjective attractiveness rating, *ObAtt* objective attractiveness rating, *SocAnx* social anxiety symptoms, *Depress* depressive symptoms, *LifeSat* life satisfaction, *EatPath* eating pathology symptoms, *T1* time --, *T2* time 2, *T3* time 3, *T4* time 4

^**^*p* < 0.001, ^*^*p* < 0.05
